# Un naevus plantaire congénital de taille moyenne: à propos d’un cas

**DOI:** 10.11604/pamj.2016.25.149.10840

**Published:** 2016-11-11

**Authors:** Youssouf Fofana, Koureissi Tall

**Affiliations:** 1 Service de Dermatologie, Centre National d’Appui à la Lutte contre la Maladie, Bamako, Mali

**Keywords:** Naevus, plantaire, enfant, Nevus, plantar, child

## Image en médecine

Le nævus (ou grain de beauté) est une forme de tumeur cutanée, développée au dépend des mélanocytes, cellules responsables de la pigmentation de la peau. On parle de naevus congénital quand il est présent dès la naissance. Avant l’adolescence, les naevus sont régulièrement distribués sur le corps avec des naevus larges (> 5 mm) peu nombreux mais prédominant alors sur le tronc (rôle d’expositions solaires brutales) alors que ceux de la face et des membres, chroniquement exposés à des doses modérées d’UV sont plus nombreux et petits. Nous rapportons le cas d'un patient âgé de 4 ans, sans antécédents médico-chirurgicaux connus, présentant une plaque pigmentée sur la plante du pied droite à la naissance. A l’inspection, nous observons une plaque pigmentée verruqueuse en carte de géographie non prurigineuse s’étendant du talon au petit et gros orteil mesurant 15 x 6 cm et dont la surface plantaire couverte est estimée à 80%. Nous avions évoqué trois hypothèses diagnostiques: un naevus le plus probable, la tache bleu mongolique, un mélanome. Une biopsie a été réalisée et l’histologie a conclu à un naevus. Le risque de transformation du naevus de taille moyenne en mélanome étant très faible, un suivi clinique sera effectué tous les 6 mois.

**Figure 1:**
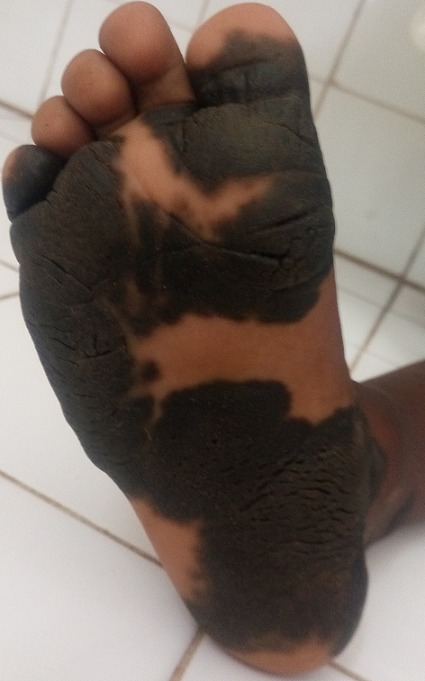
Naevus plantaire droit chez un enfant de 4 ans

